# Claudin‐2 promotes colorectal cancer growth and metastasis by suppressing NDRG1 transcription

**DOI:** 10.1002/ctm2.667

**Published:** 2021-12-29

**Authors:** Mingtian Wei, Yaguang Zhang, Xuyang Yang, Pingfan Ma, Yan Li, Yangping Wu, Xiangzheng Chen, Xiangbing Deng, Tinghan Yang, Xiaobing Mao, Lei Qiu, Wenjian Meng, Bo Zhang, Ziqiang Wang, Junhong Han

**Affiliations:** ^1^ Department of Gastrointestinal Surgery Frontiers Science Center for Disease‐related Molecular Network and State Key Laboratory of Biotherapy West China Hospital Sichuan University Chengdu China; ^2^ Research Laboratory of Tumors Epigenetics and Genomics Department of General Surgery Frontiers Science Center for Disease‐related Molecular Network State Key Laboratory of Biotherapy and Cancer Center West China Hospital Sichuan University Chengdu China; ^3^ Department of Respiratory and Critical Care Medicine State Key Laboratory of Biotherapy West China Hospital Sichuan University Chengdu China; ^4^ Department of Clinical Research Management West China Hospital Sichuan University Chengdu China; ^5^ Department of Liver Surgery & Liver Transplantation Center West China Hospital Sichuan University Chengdu China

**Keywords:** CLDN2, colorectal cancer, NDRG1, progression, ZONAB

## Abstract

Colorectal cancer (CRC) is one of the most common malignant tumours, with multiple driving factors and biological transitions involved in its development. Claudin‐2 (CLDN2), a well‐defined component of cellular tight junction, has been indicated to associate with CRC progression. However, the function of CLDN2 and the underlying mechanism whereby the downstream signalling transduction is regulated in CRC remains largely unclear. In this study, we demonstrated that CLDN2 is upregulated in CRC samples and associated with poor survival. And CLDN2 depletion significantly promotes N‐myc downstream‐regulated gene 1 (NDRG1) transcription, leading to termination of the CRC growth and metastasis in vitro and in vivo. Mechanistically, this process promotes CLDN2/ZO1/ZONAB complex dissociation and ZONAB shuttle into nucleus to enrich in the promoter of NDRG1. Thus, this study reveals a novel CLDN2/ZO1/ZONAB‐NDRG1 axis in CRC by regulating the expression of EMT‐related genes and CDKIs, suggesting CLDN2 may serve as a promising target for CRC treatment.

## Graphical abstract

1. CLDN2/ZO1/ZONAB‐NDRG1 axis in CRC regulate EMT and cell proliferation
2. Downregulation of CLDN2 promotes ZONAB relocation to nucleus and NDRG1 transcription2<egulation of CLDN2 promotes ZONAB relocation to nucleus and ND

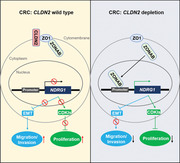

## 1 INTRODUCTION

Colorectal cancer (CRC) is one of the most common malignant tumours, with an increasing incidence in eastern countries.^1^ In the developmental process of colorectal cancer, multiple driving factors and biological behaviour transitions are involved. Epithelial–mesenchymal transition (EMT) is characterised by downregulation of epithelial phenotype molecules such as E‐cadherin and upregulation of mesenchymal phenotype molecules including Vimentin, Collagen I and Fibronectin. And it accounts for one of the key biological behaviours which is well known to facilitate migration and invasion of tumour cells.^2^, ^3^, ^4^

Claudin‐2 (CLDN2), as a component of cellular tight junction, is reported to participate in the progression of various cancers through its expression aberrance.^5^, ^6^, ^7^, ^8^ In colon, robust increase of CLDN2 expression is initially noticed in inflammatory bowel disease or CRC tissues.^9^, ^10^ And recent evidence has indicated CLDN2 as an oncogene modulating colon cancer proliferation and migration/invasion via EGFR‐mediated signalling transactivation.^11^ Although, the partial function and upstream regulatory pathways of CLDN2 have been elucidated,^11^, ^12^ its precise function and downstream targets/partners still remain largely unsolved.

N‐myc downstream‐regulated gene 1 (NDRG1) is a highly conserved cytoplasmic protein, which is well‐reported to function as a metastasis suppressor.^13^ Recent evidence indicated that NDRG1 exerted its favourable function by modulating a trail of tumour progression associated signalling pathways in multiple human cancers.^14^, ^15^, ^16^ Furthermore, a most recent investigation confirmed that NDRG1 inhibited EMT biological behaviour by interacting with caveolin‐1 and promoting its ubiquitylation in CRC.^17^

In this study, we found that CLDN2 expression was significantly elevated in CRC patients and was also closely related to tumour metastasis and patient survival. We further revealed remarkable NDRG1 upregulation in *CLDN2* knocked‐out CRC cells through RNA‐sequencing and western blot. In addition, we demonstrated that CLDN2 upregulation inhibited NDRG1 expression, resulting in epithelial–mesenchymal transition (EMT) activation and decreased expression of cyclin‐dependent kinase inhibitors (CDKIs). *CLDN2* knockdown also promoted CLDN2/ZO1/ZONAB complex dissociation, resulting in ZONAB relocation to nucleus. Thus, we uncovered a novel mechanism of CRC growth and metastasis regulated by CLDN2/ZO1/ZONAB‐NDRG1 axis, indicating CLDN2/ZO1/ZONAB‐NDRG1 axis could serve as a promising therapeutic target for CRC.

## 2 MATERIALS AND METHODS

### 2.1 Cell culture

Human colorectal cancer cell lines including Lovo, SW480, SK‐CO15, HCT116, LIM1215, SW620, Caco2, HT29, SW480, NCM460 and HEK293T (purchased from the cell bank of the Chinese Academy of Science, Shanghai, China) were routinely cultured in Dulbecco's modified Eagle's medium (DMEM) at 37°C in a humidified atmosphere of 5% CO_2_. All culture media contained 10% foetal bovine serum (FBS), 100 units/ml of penicillin and 100 μg/ml of streptomycin. All cell lines were tested and treated to exclude mycoplasma contamination.

### 2.2 Clinical sample acquisition and IHC analysis

The sample of human primary colorectal tumours, paired adjacent normal mucosa and liver metastasis tissues were obtained from the West China Hospital (Chengdu, China). All patients signed informed written consent with the approval of the Biological and Medical Ethics Committee of West China Hospital. Tissue samples were ground for western blot or fixed in 4% formalin and embedded in paraffin to obtain serial sections of 3–5 μm thick for immunohistochemical analysis (IHC) staining. Two different tissue microarrays from Shanghai Outdo Biotech Co., Ltd. comprising 104 CRC tumours and 85 adjacent normal mucosa were analysed through IHC to evaluate protein expression. Immunoreactivity score was semiquantitatively calculated based on the formula: (1 × % of weak staining) + (2 × % moderate staining) + (3 × % marked staining). The staining intensity for each sample was scored as 0 (negative), 1 (low), 2 (moderate) and 3 (high). Low and high expression of CLDN2 was based on the staining intensity for each sample (low or negative: scored as 0 and 1, high or positive: scored as 2 and 3).

### 2.3 Western blot

Western blot was performed as described previously.^18^ Briefly, after preparation in cell lysis buffer containing protease inhibitors, protein lysates (usually 20 μg) were fractionated by 8% or 10% sodium dodecyl sulphate polyacrylamide gel (SDS‐PAGE) and incubated with the primary antibody (Table S2) at a diluted concentration based on manufacture's protocol. β‐Actin, GAPDH or H3 were used as controls. And the quantifications of bands were conducted by image J.

### 2.4 Plasmid construction for CRISPR‐Cas9 knockout and overexpression

Guide RNA oligos of *CLDN2/NDRG1* (Table S3) were cloned into plenti‐CRISPRv2 and transfected into HEK293T cells, along with packing plasmid pXPAX2 and pMD2G to package *CLDN2/NDRG1* knockout virus. CRC cells were infected with above virus for 48 h, then selected with media containing 2.5 μg/ml puromycin until the density of positively infected cells reached 80–90%.

Human *CLDN2/NDRG1/ZONAB* genes were amplified by PCR and cloned into vector plenti‐CMV‐EGFP. The recombinant plasmid plenti‐CMV‐GFP‐*CLDN2/NDRG1/ZONAB* was transfected into CRC cell lines with PEI. A (GlyGlySer)_4_ linker of 12 AA was carried within the plasmids. All plasmid constructs were confirmed through DNA sequencing before use and expression efficiency was confirmed by immunofluorescence and western blot.

### 2.5 CCK8 and colony formation assays

Cell proliferation was tested with Cell Counting Kit 8 (CCK‐8, Dojindo, Kumamoto, Japan). Proper CRC cells (5000 per well in CLDN2 knockout/overexpression or vector control and its rescue assays, and 10 000 in NDRG1 knockdown/overexpression or vector control) were seeded in each well of 96‐well plates for triple duplicates. According to the manufacture's recommendation, the absorbance of mixed media was measured using a spectrophotometer (Bio‐Rad, Hercules, California, USA) at 0, 24, 48 and 72 h.

CRC cells with CLDN2/NDRG1 knockout or overexpression or vector control (2000 per well in CLDN2 and its rescue assays and 5000 in NDRG1) were seeded in the 6‐well plates and incubated at 37°C in humidified incubator for 2 weeks. Cell colonies were fixed with formalin and then stained with crystal violet. The total number of cell colonies was calculated at the end of each experiment.

### 2.6 Migration and invasion assays

Migration and invasion ability was analysed using Boyden chamber (Corning Incorporated) migration assay. For invasion assay, the membrane of the upper chamber was coated with Matrigel–medium (1:3) mixture. After starvation incubation without FBS for 12–24 h, proper CRC cells (30 000 per well in CLDN2 knockout/overexpression or vector control and its rescue assays and 50 000 in NDRG1 knockdown/overexpression or vector control) were seeded per chamber in the 24‐well plates. And 600 μl of medium containing 10% FBS was added in the lower chamber and 100 μl of sole medium was added in the upper chamber. For migration assay, no Matrigel was used and 10% FBS was added in both chambers. The migratory or invasive cells on the lower surface of the filter were fixed in 4% formaldehyde after 24–48 h of incubation and stained with haematoxylin. The cells was photographed and counted using a light microscope (200 μm, Olympus, Japan).

### 2.7 Immunofluorescence staining

CRC cells in 6‐well plates were washed with PBS, fixed with 4% paraformaldehyde and permeabilised with 0.1% Triton X‐100 in PBS. Cells were incubated with primary CLDN2 antibody, followed by EGFP‐conjugated secondary antibody incubation and counterstained with DAPI. Zeiss fluorescence microscope was used for image fluorescence captured.

### 2.8 RNA‐sequencing

CRC cells were lysed in Trizol Reagent (Invitrogen, USA) and total RNA was extracted according to the manufacturer's recommendation. Novogene Bioinformatics Technology Co. (China) performed the library preparation and RNA sequencing. Briefly, after RNA quantification and qualification, 3 μg of RNA was collected as input material. Sequencing libraries were generated using NEBNext® UltraTM RNA Library Prep Kit for Illumina® (NEB, USA) according to the manufacturer's protocols. Poly‐T oligo‐attached magnetic beads were used for mRNA purification from total RNA. The first‐ and second‐strand cDNAs were synthesised using random hexamer primer and M‐MuLV Reverse Transcriptase (RNase H‐) and DNA polymerase I and RNase H, respectively. Remaining overhangs were converted into blunt ends via exonuclease/polymerase activities.

Library fragments were purified with AMPure XP system (Beckman Coulter, USA). The quality of purified PCR products was assessed on the Agilent Bioanalyzer 2100 system. After cluster generation by TruSeq PE Cluster Kit v3‐cBot‐HS (Illumia), sequencing was performed on an Illumina Hiseq 2000/2500 platform.

RNA‐Seq reads were mapped to reference genome using TopHat v2.0.9, which used Bowtie v2.0.6 for Index of the reference genome building. The reads numbers were counted by HTSeq v0.6.1. And the gene RPKM was calculated according to the its length and the mapped reads counting. Differential expression analysis of CLDN2 knockout/overexpression or vector control was performed using the DESeq R package (1.10.1). The Benjamini and Hochberg's approach was used for *p* values adjustment with the aim to control the false discovery rate . The adjusted *p* value < .05 indicated gene expressed differentially.

### 2.9 Xenograft assay

For the tumourigenesis model, 4‐ 5‐week‐old female BALB/c nude mice were injected subcutaneously with 5 × 10^6^ HCT116 cells containing CLDN2 overexpression or vector control and Caco2 cells with CLDN2 knockout or CLDN2 and NDRG1 both knockout or control. Tumour growth was examined in 2 days’ interval, and tumour volume was calculated using the formula length (cm) × width (cm) × width (cm) × 0.5.^19^ Each mouse was sacrificed 3 weeks’ post‐injection and the tumours were dissected and weighed. For the liver metastasis model, 4‐ to 5‐week‐old female BALB/c nude mice were injected sub‐spleen with 2 × 10^6^ HCT116 cells containing CLDN2 overexpression or vector control, or Caco2 cells with CLDN2 knockout or CLDN2 and NDRG1 both knockout or control. The livers were checked for tumour metastasis and tumours were numbered 2–4 weeks’ post‐injection. Animal experiments was designed and carried out based on the standard guideline of Institutional Animal Care and Use Committee (IACUC). Tissue samples were fixed in 4% formalin and embedded in paraffin to obtain serial sections of 3–5 μm thick for immunohistochemical analysis (IHC) staining.

### 2.10 Bioinformatics analysis

mRNA expressions comparing human normal colon tissues and paired colorectal tumour tissues were obtained via NCBI mRNA database GEO profile dataset (GDS2947). Differential expression was calculated with *t*‐test. Pearson's correlation analysis of *CLDN2* and *NDRG1* mRNA levels in each case was performed using preliminary expression. Survival data in CRC were obtained from TCGA survival database Oncolnc (http://www.oncolnc.org/). Kaplan–Meier test was conducted for overall survival analysis.

### 2.11 Immunoprecipitation and chromatin immunoprecipitation assays

Immunoprecipitation (IP) and chromatin immunoprecipitation (ChIP) assays were conducted as described previously.^20^ Briefly, EGFP‐tagged CRC cells with or without depletion of *CLDN2* were harvested in lysis buffer and centrifuged. The cell lysates were incubated with anti‐GFP/ZONAB beads at 4°C overnight. The beads were washed and analysed with western blot analysis for GFP/NDRG1/ZO1 expression. For ChIP assay, cells were fixed with 1% formaldehyde for 20 min, then quenched with 125 mM glycine. Cell extracts were prepared by mechanical sonication and protein–DNA complexes were immunoprecipitated using indicated antibodies. Immunoprecipitated DNA was analysed through real‐time PCR with primers specific for NDRG1 promoter region (Table S2).

### 2.12 Quantitative PCR analysis

Total ChIP‐DNA was generated with by mechanical sonication and protein–DNA complexes were immunoprecipitated using IgG and ZONAB antibodies. The quantitative real‐time PCR amplification was started with initial denaturation at 95°C for 3 min, followed by 40 cycles of 95°C for 30 s and 60°C for 1 min. The primers specific for NDRG1 promoter region were listed in Table S2.

### 2.13 Statistical analysis

Statistical analysis was performed using Graphpad Prism 6.0 for Mac. Student's *t*‐test and chi‐square test were used for comparison of differences where appropriate. For cancer‐specific outcome analysis, Kaplan–Meier survival curves were used. Correlation between *CLDN2* and *NDRG1* mRNA levels was calculated using Pearson's correlation analysis. *p* Value less than .05 was considered as significance.

## 3 RESULTS

### 3.1 CLDN2 is upregulated in colorectal cancers and associated with poor survival

**FIGURE 1:**
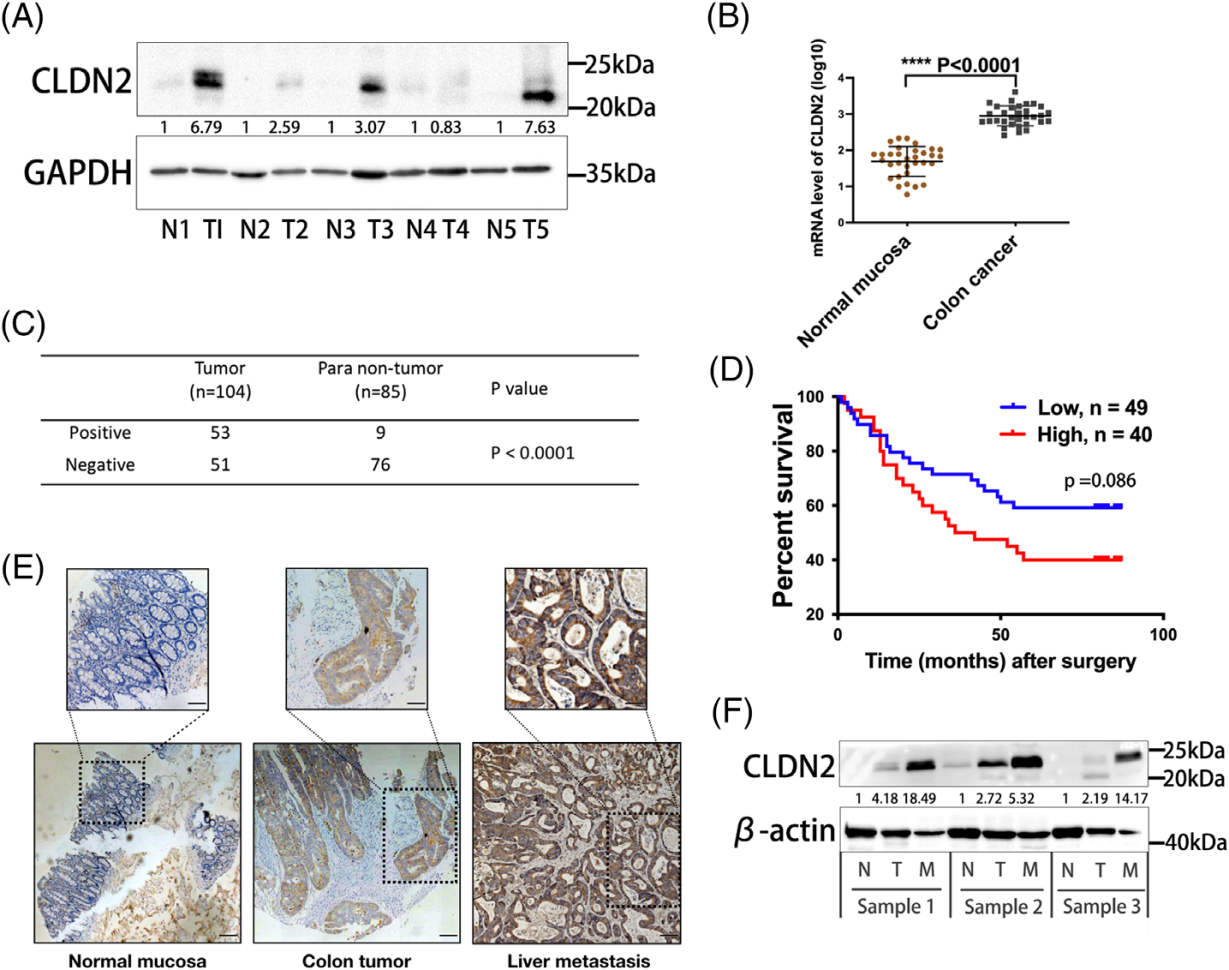
CLDN2 is upregulated in colorectal cancers and associated with poor survival. (A) Representative band of CLDN2 levels in five paired colorectal cancer and adjacent normal tissues by western blot analysis; Image J was used for bands quantification and the bands were normalised to GAPDH. (B) Relative mRNA expression of *CLDN2* in public TCGA datasets (GDS2947); *p* value was calculated with *t*‐test. (C) Statistical analysis of CLDN2 IHC expressions from 189 paraffin‐embedded human colorectal cancer tissues; *p* value was calculated with chi‐square test. (D) Overall survival of 89 colorectal cancer patients stratified by low versus high CLDN2 expression. (E) Representative image of IHC staining for CLDN2 in adjacent normal mucosa, colorectal tumour tissue and liver metastasis tissue; scale bar, 100 μm (lower panel), 50 μm (upper panel). (F) Representative band of CLDN2 levels in three paired normal mucosa, colorectal cancer and liver metastasis tissues by western blot analysis; Image J was used for bands quantification and the bands were normalised to β‐Actin

CLDN2, a component of cellular tight junction, is reported to participate in the progression of various cancers through its expression aberrance.^5^, ^6^, ^7^, ^8^ To investigate the potential role of CLDN2 in colorectal cancer development, the expression of CLDN2 was examined in five paired colon cancer and adjacent normal tissues, which revealed that most of CRC tissues showed remarkable high expression of CLDN2 compared to its paired adjacent normal tissues (Figure 1A). Subsequent analysis of *CLDN2* mRNA levels in the publicly available colorectal datasets indicated a significantly elevated expression of *CLDN2* in colorectal cancers (Figure 1B). In addition, CLDN2 level was examined in 104 cases of colorectal cancer tumours and 85 cases of cancer adjacent normal tissues. According to the level of CLDN2 protein by IHC analysis, 53/104 colorectal tumour tissues positively expressed CLDN2, significantly higher than 9/85 CLDN2‐positive in adjacent normal tissues (Figure 1C). Based on the level of CLDN2 protein, Kaplan–Meier analysis of patients’ follow‐up data suggested favourable overall survival in patients with low level of CLDN2 protein, compared to those with high level of CLDN2 protein (Figure 1D, Table S1).

To further explore the role of CLDN2 in colorectal cancer progression, resected liver metastases and corresponding paired colorectal tumour tissues, along with adjacent normal mucosa was collected from same CRC patient. The sample was subjected to investigate the CLDN2 expression, and we observed the obvious increase from normal mucosa to CRC primary foci which is consistent with the results showed in Figure 1A. Intriguingly, the highest expression appeared in liver metastasis, implying that CLDN2 may be involved in liver metastasis from CRC primary foci (Figures 1E, F and S1A–C).

Collectively, these results demonstrate that CLDN2 is upregulated in human colorectal cancers and associates with poor overall survival, suggesting its oncogenic role in colorectal cancer progression.

### 3.2 CLDN2 promotes colorectal cancer cell proliferation and migration/invasion

**FIGURE 2:**
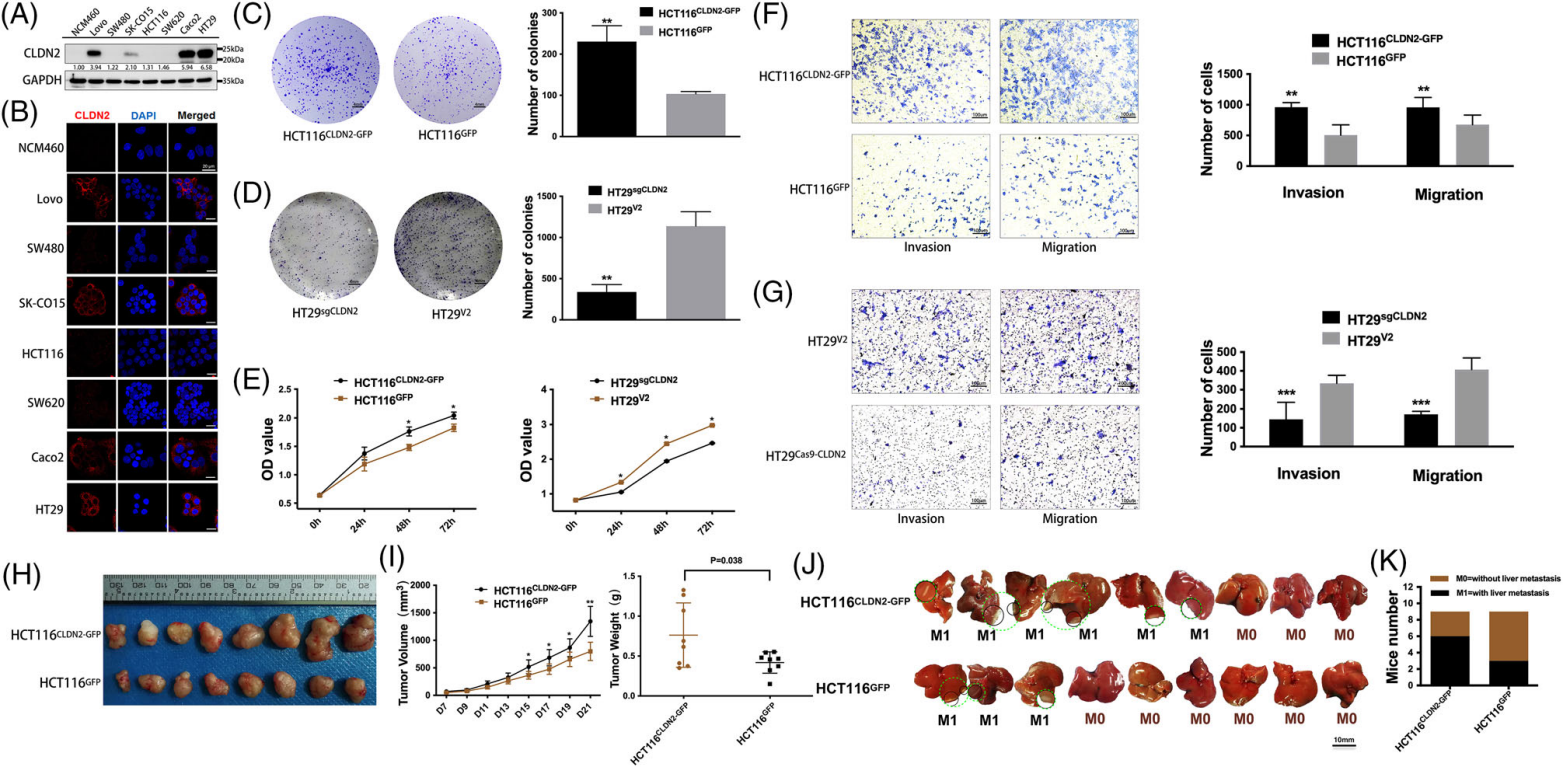
CLDN2 enhances colorectal cancer cell tumourigenesis and metastasis in vitro and in vivo. (A) Representative band of CLDN2 levels in CRC cell lines by western blot analysis; Image J was used for bands quantification and the bands were normalised to GAPDH. (B) Immunofluorescence analysis of CLDN2 in indicated colorectal cancer cell lines; left panel represents CLDN2 (red), middle panel represents nucleus by DAPI staining (blue) and right panel represents merged images (black); scale bar, 20 μm. (C, D) Representative colony formation images and quantification numbers of HCT116 and HT29 cells with either *CLDN2* overexpression or knockout; scale bar, 4 mm. (E) Representative cell proliferation of HCT116 and HT29 cells measured by CCK8 assay. (F, G) Representative transwell invasion/migration images and quantification numbers of HCT116 and HT29 cells; scale bar, 100 μm; *p < .01, **p < .01, ***p < .001 by *t*‐test. Similar results were repeated at least three times. (H) Gross photos of removed xenograft tumours 3 weeks after subcutaneously injection of HCT116 cells (*n* = 8). (I) Average weight of removed xenograft tumours; error bars indicate SD. (J) Images of liver metastases 2 weeks after injection of HCT116 cells into the spleen (*n* = 9); black circle indicates metastasis lesions. (K) Percentage of mice with and without liver metastases

To evaluate the expression of CLDN2 in human colorectal cancer cell lines, eight cell lines including NCM460 (normal colon cell), Lovo, SW480, SK‐CO15, HCT116, SW620, Caco2 and HT29 were cultured to examine CLDN2 level. Western blot indicated that a majority of colorectal cancer cell lines (4/8) positively expressed CLDN2 protein (Figure 2A). Consistent with the results detected by western blot, immunofluorescence analysis showed the expression of CLDN2 in indicated five cell lines (Figure 2B).

To identify the functional role of CLDN2 in colorectal cancer, *CLDN2* was knocked out in CLDN2‐high HT29 cells through CRISPR‐Cas9 and was overexpressed in CLDN2‐low HCT116 cells using plenti‐CMV‐EGFP plasmid. Successful *CLDN2* knockout and overexpression were confirmed using western blot analysis and GFP imaging (Figure S1D, E). In colony formation assay, we observed a significantly decreased number of colonies in CLDN2 knockout cells (HT29^sgCLDN2^), whereas the exogenous expression of CLDN2 led to increased colony forming ability in HCT116^CLDN2‐GFP^ (Figure 2C, D). This growth promoting ability of CLDN2 in colorectal cancer was further confirmed by subsequent CCK8 proliferation assay, where HT29^sgCLDN2^ cells were less proliferative than control cells (HT29^V2^) and HCT116^CLDN2‐GFP^ cells were more proliferative than control cells (HCT116^GFP^) (Figure 2E). To further determine effects of CLDN2 on CRC invasion and migration capability, cells were seeded in Boyden chamber with or without Matrigel coating, respectively. There were significantly less penetrated HT29^sgCLDN2^ cells through the membrane compared to the control HT29 cells and more penetrated HCT116^CLDN2‐GFP^ cells compared to the control HCT116 cells (Figure 2F, G). Consistently, the promoting role of CLDN2 in colorectal cancer migration/invasion was verified by wound‐healing assay with HT29 and Caco2 cells (Figure S2A, B). Together, these results indicate that CLDN2 has a significant contribution to CRC proliferation and migration/invasion.

### 3.3 CLDN2 enhances colorectal cancer cell tumourigenesis and metastasis in vivo

To validate whether CLDN2 could facilitate the growth of colorectal cancer in vivo, CLDN2‐overexpressing HCT116 and vector control cells were injected subcutaneously into BALB/c nude mice to establish human colorectal cancer xenografts. As shown in Figure 2H, I, the average weight of mouse tumours at the time of sacrifice was heavier in mice injected with CLDN2‐overexpressing HCT116 compared to mice injected with vector control (0.76 ± 0.14 g, *n* = 8 vs. 0.42 ± 0.04 g, *n* = 8, *p* = .038).

Similarly, we established a liver metastasis model by directly injecting HCT116^sgCLDN2^ and HCT116^GFP^ cells into the spleen of BALB/c nude mice (*n* = 9 for each group). All mice were sacrificed to detect liver metastasis nodules after 2 weeks. Mice injected with CLDN2‐overexpressing HCT116 cells had a higher rate of metastasis (6/9) compared to mice injected with vector control cells (3/9) (Figure 2J, K). Taken together, the results provide the insight that CLDN2 promotes colorectal cancer progression in vitro and in vivo.

### 3.4 CLDN2 suppresses NDRG1 expression in CRC

**FIGURE 3:**
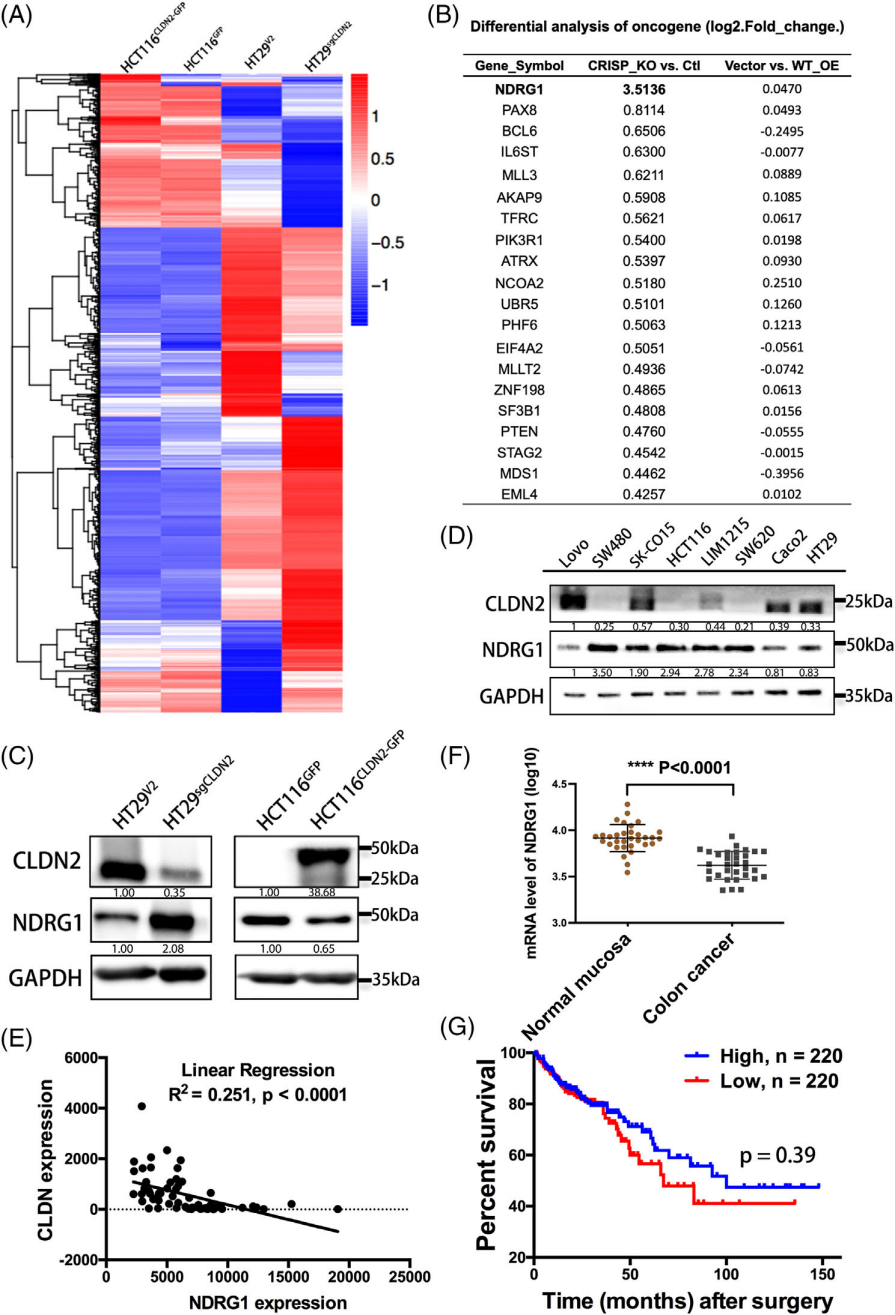
CLDN2 suppresses downstream NDRG1 expression. (A) Heatmap depiction of differentially expressed genes between *CLND2* knockout and *CLDN2* overexpression cells (*n* = 1). (B) List of top 20 tumour‐related factors, log2 fold‐change value represents that gene changes. (C) Western blot analysis of NDRG1 level in cells with either *CLDN2* knockout or *CLDN2* overexpression. (D) Relationship of CLDN2 and NDRG1 expression in eight colorectal cell lines as detected by western blot analysis. (E) mRNA quantification of co‐expression for *CLDN2* and *NDRG1* on public colorectal cancer datasets (GDS2947); *p* value was calculated with Pearson's correlation analysis. (F) Relative mRNA expression of *CLDN2* in public TCGA datasets (GDS2947); *p* value was calculated with *t*‐test. (G) Overall survival of 440 colorectal cancer patients stratified by low versus high *CLDN2* expression in public database (survival data based on mRNA expression levels at http://www.oncolnc.org/)

To uncover the underlying molecular mechanism of how CLDN2 facilitate colorectal cancer progression, we performed RNA sequencing (RNA‐seq) on CRISPR‐mediated *CLDN2* knockout cells (HT29^sgCLDN2^ and HT29^V2^) and CLDN2‐expressing cells (HCT116^CLDN2‐GFP^ and HCT116^GFP^). Figure 3A showed the heatmap of cluster analysis for differentially expressed downstream target genes in two paired cell groups. We totally identified 54 differentially expressed tumour‐related genes from comparison between CRISPR‐mediated *CLDN2* knockout cell and vector control cell. Among these genes, *NDRG1* ranked the highest fold change (3.51 for log2 fold‐change, Figure 3B).

To determine whether NDRG1 was indeed regulated by CLDN2, we performed western blot analysis in *CLDN2*‐knockout HT29 and CLDN2‐expressing HCT116 cell lines, along with their vector controls. The results clearly showed negatively correlated expression levels of CLDN2 and NDRG1, indicating that CLDN2 modulated the protein expression of NDRG1 (Figure 3C). In addition, eight colorectal cancer cell lines as we mentioned in Figure 2A were utilised to detect the levels of CLDN2 and NDRG1 by western blot analysis. The results consistently identified relatively low expression of NDRG1 in high CLDN2‐expressing cells (Lovo, SK‐CO15, LIM1215, Caco2 and HT29) and high expression of NDRG1 in low CLDN2‐expressing cells (SW48, HCT116 and SW620) (Figure 3D). To further investigate the correlation between CLDN2 and NDRG1, mRNA quantification analysis of *CLDN2*/*NDRG1* co‐expression revealed negative linear correlation on public colorectal cancer datasets (*R* ^2^ = .251, *p* < .0001) (Figure 3E). The mRNA levels of *NDRG1* in the same GEO Profiles (GDS2947) indicated significantly decreased expression of NDRG1 in colorectal cancers (Figure 3F). As for prognosis, based on an oncogenic database analysis, compared with patients with high expression of *NDRG1* mRNA, 220 CRC patients with low expression of *NDRG1* mRNA suffered shorter overall survival time (Figure 3G). The overall survival of CRC patients showing in Figures 1D and 3G strongly implies that the reverse correlation between CLDN2 and NDRG1 has a significant role in CRC development and prognosis.

### 3.5 NDRG1 suppresses CRC cell proliferation and migration/invasion

**FIGURE 4:**
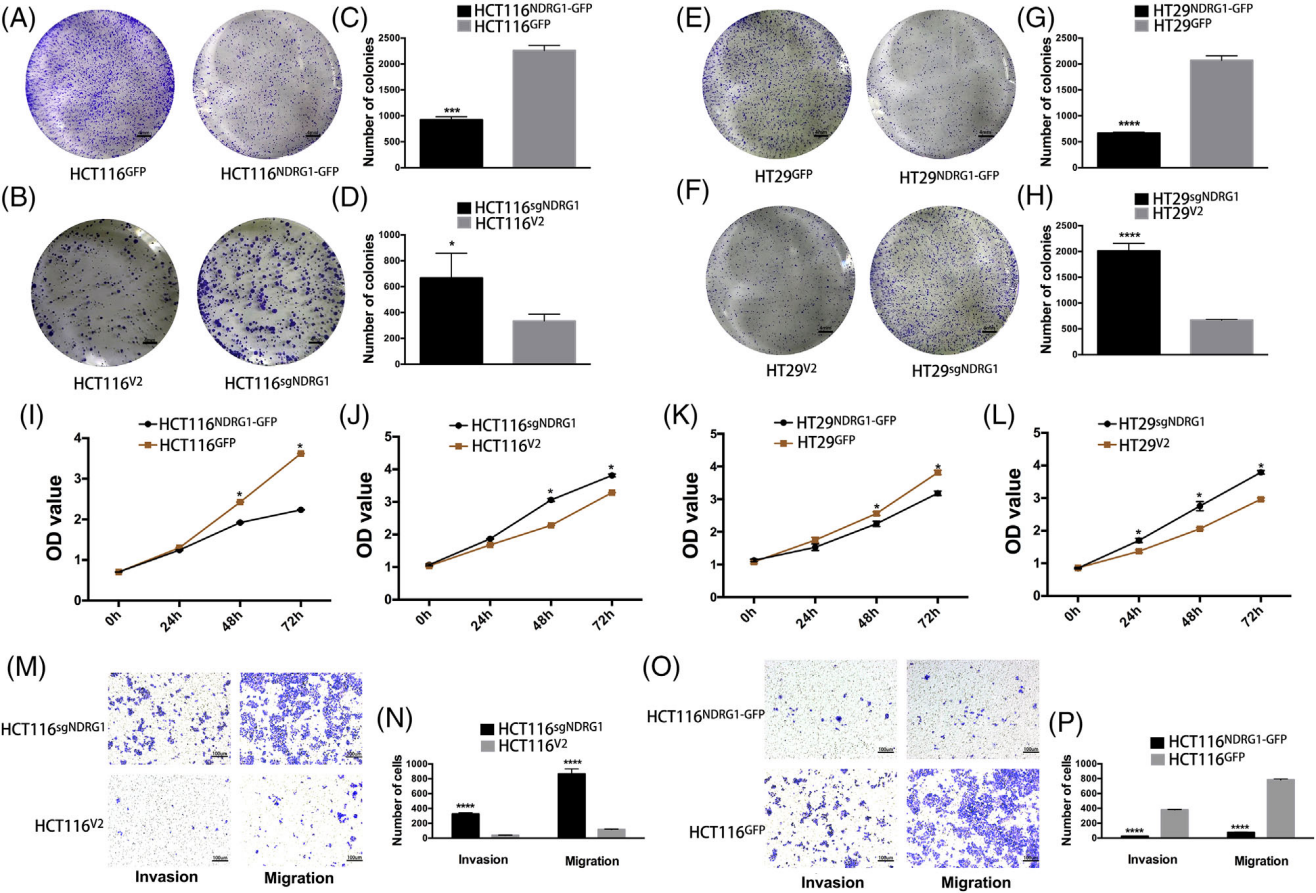
NDRG1 suppresses colorectal cancer cell proliferation and migration/invasion. (A–H) Representative colony formation images and quantification numbers of HCT116 and HT29 cells either overexpressing *NDRG1* or knocking out *NDRG1*; scale bar, 4 mm. (I, J) Representative cell proliferation of HCT116 and HT29 cells either overexpressing *NDRG1* or knocking out *NDRG1* measured by CCK8 assay. (M–P) Representative transwell invasion/migration images and quantification numbers of HCT116 cells either overexpressing *NDRG1* or knocking out *NDRG1*; scale bar, 100 μm; *p < .01, **p < .01, ***p < .001, ****p < .0001 by *t*‐test. Similar results were repeated at least three times

We next investigated the potential function of NDRG1 in colorectal cancer progression. Using CRISPR‐Cas9 and recombinant expression plasmid, *NDRG1* knockout and *NDRG1* overexpression in HT29 and HCT116 cell lines were established respectively and NDRG1 expression was confirmed by western blot analysis (Figure S1F, G). As expected, exogenous expression of NDRG1 led to decreased colony forming ability (colony formation assay) and proliferation (CCK8 assay). However, knockout of *NDRG1* in HT29 and HCT116 cells elevated the proliferation ability of colorectal cancer cells (Figures 4A–L and S1G). As for migration and invasion assays, CRISPR‐mediated *NDRG1* knockout in HCT116 cells induced a significantly stronger migration and invasion property, compared with vector control cells. On the contrary, overexpression of NDRG1 significantly reduced the number of HCT116 cells penetrated through the Boyden chamber membrane after 72 h (Figure 4M–P). The result that NDRG1 inhibits colorectal cancer cell proliferation and migration/invasion, partially provides evidence to support the involvement of NDRG1 in CLDN2‐mediated colorectal cancer progression.

### 3.6 NDRG1 is essential for CLDN2‐mediated CRC cell proliferation and migration/invasion in vivo and in vitro

**FIGURE 5:**
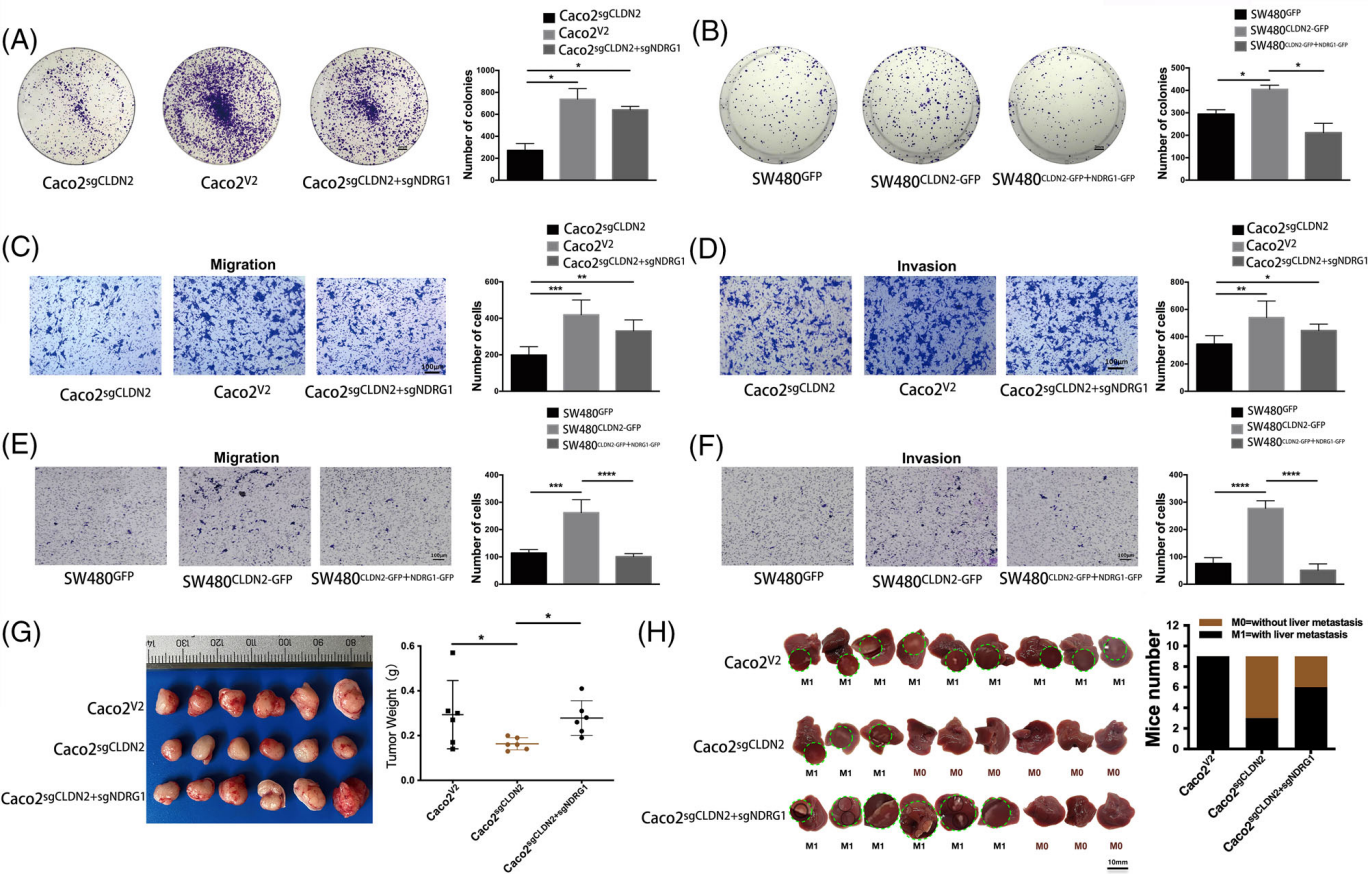
NDRG1 is essential for CLDN2‐mediated CRC cell proliferation and migration/invasion in vivo and in vitro. (A, B) Representative colony formation images and quantification numbers of Caco2 and SW48 cells either knocking out *NDRG1* in CLDN2 knockout or overexpressing *NDRG1* in CLDN2 overexpression; scale bar, 3 mm. (C–F) Representative transwell invasion/migration images and quantification numbers of Caco2 and SW48 cells either knocking out *NDRG1* in CLDN2 knockout or overexpressing *NDRG1* in CLDN2 overexpression; scale bar, 100 μm; *p < .01, **p < .01, ***p < .001, ****p < .0001 by *t*‐test. Similar results were repeated at least three times. (G) Gross photos and average weight of removed xenograft tumours 3 weeks after subcutaneously injection of Caco2 cells (*n* = 6); *p < .01 by *t*‐test. (H) Images and percentage of mice with and without liver metastases after injection of Caco2 cells into the spleen (*n* = 9); black circle indicates metastasis lesions. M0: without metastasis, M1: liver metastasis

To verify the regulatory role of CLDN2 and NDRG1 in CRC, we performed the rescue experiments by decreasing the NDRG1 expression in CLDN2 knockout cell and inducing the NDRG1 expression in CLDN2 overexpressing cell. As expected, knockout of *NDRG1* in CLDN2‐absence context led to rescue the proliferation ability of CRC investigated by colony formation assay (Figures 5A and S2C), while exogenous expression of NDRG1 in CLDN2‐overexpressing cell decreased the proliferation ability (Figure 5B). As for in vitro metastasis model, *NDRG1* knockout in CRC cells rescued the migration and invasion abilities of CLDN2 knockout cells (Figures 5C, D and S2D). On the contrary, overexpression of both CLDN2 and NDRG1 incurred significant reduction of cells penetrated through the Boyden chamber membrane (Figure 5E, F). Moreover, we generated in vivo mouse xenograft model and found that knockout of *NDRG1* in CLDN2‐absence context resulted in bounce back of the subcutaneous tumour size from shrank (Figure 5G). Meanwhile, we also observed more liver metastasis (6/9 mice) in both *CLDN2* and *NDRG1* knockout group compared with single CLDN2 knockout group (3/9 mice), while 9/9 mice showed liver metastasis in control group (Figure 5H). More importantly, most of positive liver metastasis in *CLDN2* and *NDRG1* knockout mice (5/6) had multiple metastatic lesion (Figure 5H). Together, these results suggest that the repression of NDRG1 expression is involved in CLDN2‐mediated CRC progression and invasion.

### 3.7 CLDN2‐mediated NDRG1 suppression modulates colon cancer cell behaviours by activating EMT and decreasing CDK inhibitor expression

**FIGURE 6:**
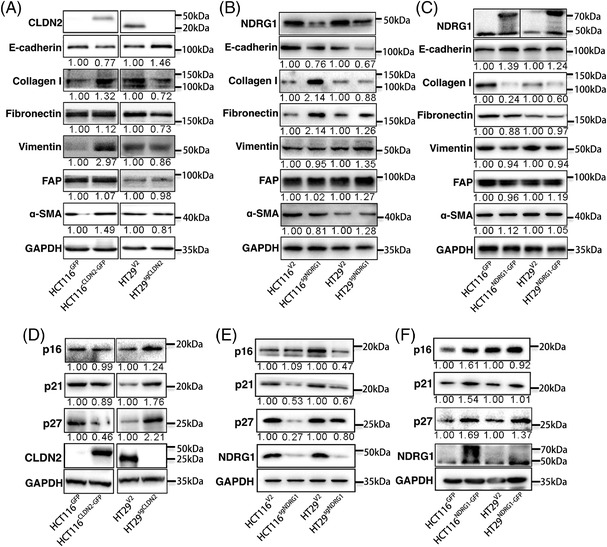
CLDN2‐mediated NDRG1 suppression modulates colon cancer cell behaviours by activating EMT and increasing CDK inhibitor expression. (A) Expression of the indicated EMT markers (E‐cadherin, Collagen I, Fibronectin, Vimentin, FAP and a‐SMA) in HCT116 and HT29 cells either overexpressing *NDRG1* or knocking out *NDRG1*. (B, C) Expression of the indicated EMT markers in cells overexpressing *NDRG1* or knocking out *NDRG1*. (D) Expression of CDK inhibitors including p16, p21 and p27 in HCT116 cells either overexpressing *CLDN2* or HT29 cells knocking out *CLDN2*. (E, F) Expression of CDK inhibitors including p16, p21 and p27 in HCT116 and HT29 cells overexpressing *NDRG1* and knocking out *NDRG1*. Similar results were repeated at least three times

Abnormal expression of E‐cadherin and parallel mesenchymal molecules are the hallmarks of epithelial–mesenchymal transition (EMT), a process involved in cancer initiation and progression.^21^ To gain insights into the mechanism whether CLDN2 regulated downstream EMT genes, we next analysed the expression profiles of EMT‐signature markers in colorectal cancer cells with either stable CRISPR‐mediated *CLDN2* knockout or *CLDN2* overexpression. Western blot analysis detected significant upregulation of epithelial cell marker E‐cadherin and accompanied with downregulation of three mesenchymal cell markers, including Collagen I, Fibronectin and Vimentin in *CLDN2* knockout cell HT29^sgCLDN2^ compared to the control cell line HT29^V2^. In contrast, overexpression of CLDN2 in colorectal cancer cells (HCT116^CLND2‐GFP^) revealed downregulation of E‐cadherin and upregulation of Collagen I, Fibronectin and Vimentin (Figure 6A). Meanwhile, a panel of cell‐cycle‐related proteins such as cyclin‐dependent kinase inhibitors (CDKIs) including p16, p21 and p27 were elevated in CLDN2‐low cells (HT29^sgCLDN2^ and HCT116^GFP^) (Figure 6D). We also examined the stemness and apoptosis‐related proteins (Oct4, Sox2, CD133, Caspase9 and Bax), which showed no significant difference in HT29 and HCT116 cell lines (Figure S3A–D).

To validate whether CLDN2‐mediated NDRG1 suppression consistently modulated EMT‐related genes in a similar manner with CLDN2, the above‐mentioned EMT markers and CDKIs were re‐evaluated in *NDRG1* knockout and overexpression cells. As expected, knockout of *NDRG1* in both HT29 and HCT116 decreased E‐cadherin and increased Collagen I, Fibronectin and Vimentin levels, as well as decreased p16, p21 and p27 levels (Figure 6B, E). Overexpression of NDRG1 in HT29 and HCT116 cells confirmed that NDRG1 expression was positively correlated to E‐cadherin, p16, p21 and p27 and negatively correlated to Collagen I, Fibronectin and Vimentin (Figure 6C, F).

**FIGURE 7:**
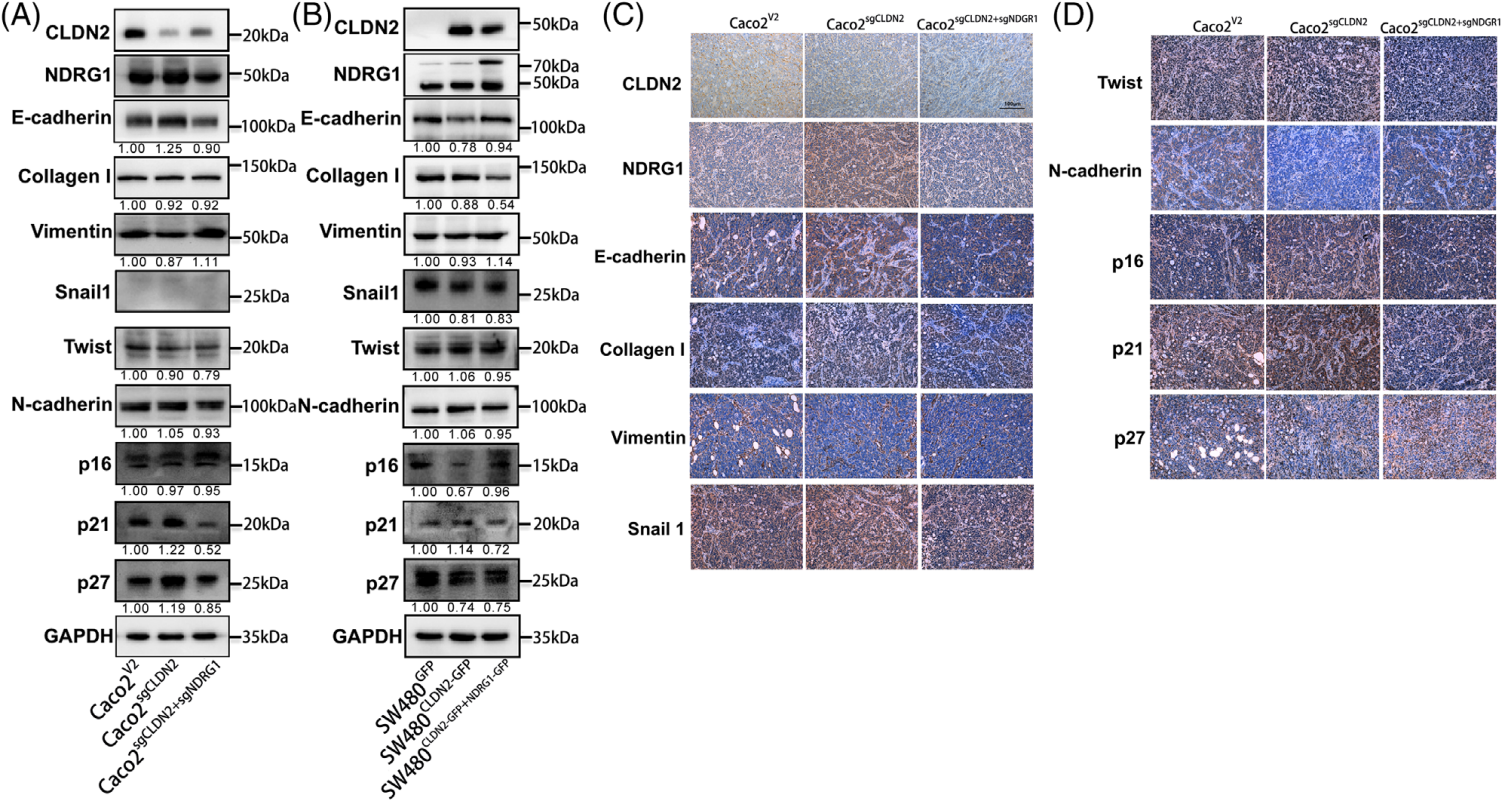
CLDN2‐mediated NDRG1 suppression modulates colon cancer cell behaviours in rescue experiments. (A, B) Western blot analysis of expression of the NDRG1, indicated EMT markers (E‐cadherin, Collagen I, Vimentin, Snail 1, Twist and N‐cadherin) and CDKs (p16, p21 and p27) in Caco2 and SW480 cells either knocking out *NDRG1* in CLDN2 knockout or overexpressing *NDRG1* in CLDN2 overexpression. (C, D) IHC analysis of representative images for above factors (CLDN2, NDRG1, E‐cadherin, Collagen I, Vimentin, Snail 1, Twist, N‐cadherin, p16, p21 and p27) in mice tumour xenografts

Moreover, in the rescue assays, knockout of NDRG1 in CLDN2‐depletion cell decreased the E‐cadherin, p21 and p27 level and increased the vimentin level (Figures 7A and S3E). The result was nearly verified by exogenous expression of NDRG1 in CLDN2 overexpression western blot (Figure 7B). IHC of mice xenograft confirmed that knockout of CLDN2 induced upregulation of NDRG1, E‐cadherin, p16 and p21 and downregulation of N‐cadherin (Figure 7C, D). Cell‐cycle profiling also confirmed that downregulation of CLDN2 would increase the rate of G0/G1, supporting the rational of cell‐cycle arrest by CDKIs (Figure S1H). IHC results in the clinical colorectal liver metastasis tissues were consistent with the protein expression detected by western blot (Figure S4). In addition, the expression of CLDN2 in colorectal liver metastasis is relatively high (Figure S5A). Taken together, CLDN2‐mediated NDRG1 suppression modulates the expression of downstream EMT markers and CDKI genes to facilitate colon cancer progression.

### 3.8 CLDN2 loss promotes CLDN2/ZO1/ZONAB complex dissociation and ZONAB shuttle into nucleus to enrich in the promoter of NDRG1

**FIGURE 8:**
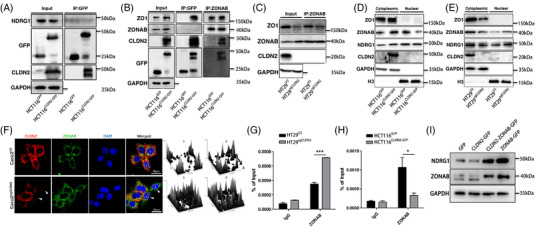
CLDN2 loss promotes CLDN2/ZO1/ZONAB complex dissociate and ZONAB shuttle into nucleus. (A) Immunoprecipitation and western blot analysis of the expression relationship between CLDN2 and NDRG1 in HCT116 cells overexpressing *CLDN2*; the band below CLDN2 may be a degradation band. (B) The interaction and correlation between CLDN2 and ZO1/ZONAB, and between ZONAB and ZO1/NDRG1 in HCT116 cells overexpressing *CLDN2* detected by Immunoprecipitation and western blot; the band below CLDN2 may be a degradation band. (C) The interaction and correlation between ZONAB and ZO1/NDRG1/CLDN2 in HT29 cells knocking out *CLDN2*. (D, E) Expression of ZO1, ZONAB, NDRG1 and CLDN2 in cytoplasmic and nuclear fraction of HCT116 and HT29 cells either overexpressing *CLDN2* or knocking out *CLDN2*. (F) Immunofluorescence analysis of the expression relationship between CLDN2 and nuclear ZONAB. The right two panels represented quantification of nuclear ZONAB in single cell marked as 1 and 2, respectively. Top 1 and 2 quantification represented wild type cells, while bottom 1 and 2 were CLDN2 depletion cells. (G, H) The enrichment of ZONAB at the promoter region of *NDRG1* in HCT116 cells overexpressing *CLDN2* or HT29 cells knocking out *CLDN2* detected by ChIP assay. (I) WB analysis of NDRG1 in CRC cells overexpressing *ZONAB*

To further determine whether CLDN2 modulates the protein expression of NDRG1 in colorectal cancer cells, we performed immunoprecipitation assay in HCT116 cells. We did not detect NDRG1 pulled down by anti‐GFP antibody, suggesting CLDN2 may indirectly modulate NDRG1 (Figure 8A). Since a previous study had confirmed the interaction between CLDN2, ZO1 and ZONAB, which was involved in human lung adenocarcinoma cell proliferation,^22^ we wondered if CLDN2 indirectly modulates the expression of NDRG1 by similarly interacting with ZO1/ZONAB in colorectal cancer cells. As expected, Co‐IP analysis indicated that only ZO1 showed interaction with CLDN2 (Figure 8B), whereas ZONAB interacts with ZO1 (Figure 8B, C). Meanwhile, decreased ZO1 along with increased ZONAB levels were detected in CLDN2‐low whole cell lysis input, revealing potential re‐distribution of ZONAB in the cytoplasm and nucleus (Figure 8B, C). In the cytoplasmic fraction, we observed lower levels of ZO1 and ZONAB in CLDN2‐low cells, whereas ZONAB level was decreased by CLDN2 expression in the nuclear fraction (Figure 8D). It is not somewhat surprising that the converse of remarks was also true in CLDN2 depletion CRC cells (Figure 8E). In the meantime, the immunofluorescence assay illustrated that cells with high expression of membrane CLDN2 contained less nuclear ZONAB than cells with low expression of membrane CLDN2, which may suggest the re‐location of ZONAB affected by CLDN2 (Figures 8F and S5B). Moreover, ZONAB ChIP‐qPCR analyses in HT29 cells showed significant ZONAB enrichment at the promoter region of NDRG1 in CLDN2 downregulation cells, while remarkable decrease in CLDN2 overexpressed HCT116 cells, indicating possible transcriptional activation of NDRG1 by ZONAB (Figure 8G, H). And this hypothesis is further supported by the results from luciferase reporter gene assay (Figure S6A). Meanwhile, we also found that overexpression of ZONAB led to the increase of NDRG1 expression (Figures 8I and S6B, C). A previous study indicated that CLDN2 contained a PDZ‐binding motif, which interacted with the PDZ domain of the scaffolding protein ZO1 on the membrane, and ZONAB shuttles between the tight junction and nucleus.^23^ Therefore, we proposed a working model that CLDN2 forms a complex with ZO1/ZONAB, once loss of CLDN2 would induce the dissociation of CLDN2/ZO1/ZONAB complex, leading to ZONAB relocation to the nucleus and enrichment at the promoter of NDRG1 to trigger its transcription, eventually inhibits CRCs growth and metastasis.

Collectively, CLDN2 stabilises CLDN2/ZO1/ZONAB complex on the membrane and prevents ZONAB shuttle into the nucleus. Meanwhile, CLDN2 depletion would promote the complex dissociation and ZONAB shuttle into nucleus to be enriched at the promoter of NDRG1, which subsequently induces NDRG1 transcription.

## 4 DISCUSSION

Although experimental and clinical studies have explored the function of CLDN2 in various cancer initiation and development processes,^23^, ^24^, ^25^, ^26^ the precise role and underlying mechanism of CLDN2 in CRC remain largely unclear. Our study provides evidence illustrating that CLDN2 promotes colorectal cancer progression by suppressing *NDRG1* expression via stabilising CLDN2/ZO1/ZONAB complex. Based on the results from clinical CRC tissues and public TCGA databases, our study revealed increased CLDN2 expression in CRC tumours compared with paired adjacent normal mucosa and predicted shorter overall survival in CLDN2‐high patients. Further with loss‐ and gain‐of‐function experiments, this study unveiled that CLDN2 positively correlated to CRC cell proliferation and migration/invasion, which was validated by xenograft tumourigenesis and liver metastasis models in vivo. Although the 1‐year survival rate is close in the CLDN2 high and low groups in Figure 1D, other confounding factors such as environmental influences, adjuvant chemotherapy or gene mutation etc. which may activate some CLDN2‐related oncogenes or inhibit antioncogenes during the subsequent development of disease in the two groups would affect patient mortality. Similarly, although we found no obvious correlation between the CLDN2 expression level and the aggressiveness of different colon cell lines, a few trend of more metastasis and lymphatic vascular/perineural invasion in high level of CLDN2 compared to that in low level of CLDN2 (metastasis: 7.5% vs. 2.0%; invasion: 5% vs. 2.0%, Table S1) were evaluated in our clinical samples. The reason may lie on the relatively small sample size or the nature of heterogeneity in every single patient. Collectively, our study confirms the facilitating role of CLDN2 in CRC development and progression.

This study sheds light on the first mechanistic insight into CLDN2‐regulated NDRG1 expression in colorectal cancer. According to RNA‐sequencing analysis, we first identified a ranking list of downstream genes differentially regulated by CLDN2. Combining the essential role of NDRG1 in cancer metastasis inhibition,^14^, ^15^, ^16^ we sequentially validated its negative correlation with CLDN2 expression as indicated by western blot and Pierson correlation analyses. In line with previously reported results,^27^ our observations indicated that NDRG1 downregulation in colorectal cancer tissue was involved in tumour initiation and progression by a train of NDRG1‐knockout and NDRG1 overexpression‐based colorectal cell function assays. Furthermore, the consistent oncogenic roles of CLDN2 upregulation and NDRG1 downregulation suggested a negative regulation of CLDN2 on NDRG1 expression.

As is well reported, NDRG1 plays its contradictory role in numerous cancers progression via various signalling pathways.^28^, ^29^, ^30^ NDRG1 is widely supported as a suppressor in prostate cancer, CRC and breast cancer.^14^, ^15^, ^31^, ^32^ For example, Dong and his colleagues combined their data with TCGA database, showing that NDRG1 regulated EMT‐associated protein expression (Twist1, Snail, VE‐cadherin and vimentin decreased, whereas E‐cadherin increased) in gastric carcinoma, which is negatively correlated with poor prognosis.^33^ And it could inhibit EMT by binding to membrane‐associated E cadherin and β‐catenin, which forms a complex to inactivate related signalling pathways.^15^, ^34^ The latest research also demonstrated that NDRG1 acted its suppressing role via downregulating EGFR through the EGFR inhibitor MIG6.^35^ Furthermore, NDRG1 overexpression inhibited glioblastoma cell growth by decreasing expression of cyclin D1/E and CDK 2/4 in a cell‐cycle signalling pathway.^36^ Thus, some reports suggested NDRG1 as a poor prognostic predictor in prostate cancer^37^ and inflammatory breast cancer.^38^ However, other studies implied that NDRG1 may functions in tumour microenvironment remodelling.^39^, ^40^ A recent research on the microenvironment development of hepatocellular carcinoma (HCC) illustrated NDRG1 increase in HCC tissues, which cooperated with FOXQ1 to promote hepatic stellate cells recruitment. The findings discovered a facilitating role of FOXO1/NDRG1 axis which acted as a downstream factor of cancer‐associated fibroblasts.^41^ Similar stimulative effect of NDRG1 was also observed in prostate cancer with regard to the EMT process.^42^ However, NDRG1 potentially reduced GLI1, a key driver of pancreatic cancer, to suppress cell migration mediated by pancreatic stellate cells.^29^ Therefore, it is apparent that NDRG1 functions probably rely on a specific context in different cancer types.

In our study, we first revealed that downregulation of CLDN2 and upregulation of its downstream NDRG1 achieved similar EMT and CDKIs regulation in CRC, which was confirmed by the rescue experiments in another CRC cell line. This result is consistent with previous findings that low expression of CLDN2 is companied with increasing E‐cadherin and decreasing Vimentin in CRC cells, which would suppress cell proliferation and EMT.^43^ And Paquet‐Fifield et al. also confirmed its self‐renewal role within CRC stem‐like cells in a CLDN2‐mediated regulation of YAP activity and miR‐222‐3p expression way.^44^ However, Dan et al. reported a converse result that CLDN2 silencing by itself induced elevated SMA and upregulated Slug in tubular cell in a RHOA‐dependent manner.^45^ The repressive role of CLDN2 on EMT may partly attribute that initial CLDN2 elevation in response to prolonged inflammation could support a switch from a proliferative to a pro‐fibrotic state, which was crucial during epithelial regeneration. And prolonged cytokines may not only induce CLDN2 loss but also affect other factors to suppress EMT. Taken together, these results provide adequate evidences to support a regulatory pathway from the upstream CLDN2 and its modulating gene NDRG1 to the downstream EMT and CDKIs in CRCs. And the EMT process induced by CLDN2 accounts for the critical step of distal metastasis in CRCs. Our results partly coincide with that in one most recent study.^46^ The research identified that CLDN2 is critical for promoting CRC liver metastasis in the established PDX model. Though IHC analysis of clinical primary tumour and paired liver metastasis both showed high expression of CLDN2 without statistical difference between groups, patients with increased CLDN2 levels in primary tumour developed more with liver metastasis within 5 years. In agreement with these findings, we recommend CLDN2 may serve as a prognostic biomarker and targeted therapeutic agents for CRC patients.

As reported, CLDN2 forms a complex with the scaffolding protein ZO1 on the membrane. And ZO1 interacts with ZO1‐associated nucleic acid binding protein (ZONAB) via its SH3 domain in the cytoplasm, forming a complex CLDN2/ZO1/ZONAB. As a Y‐box transcription factor, ZONAB can shuttle from the tight junction to the nucleus and its nuclear accumulation promotes the expression of cell‐cycle‐related genes. In our study, we found that CLDN2 loss in the membrane would cause dissociation of the CLDN2/ZO1/ZONAB complex, setting ZONAB free to shuttle into the nucleus to regulate the expression of proliferation‐ and migration/invasion‐related genes. The similar phenomenon is also observed in lung adenocarcinoma A549 cells investigated by Ikari et al. Their group observed that the promoting role of CLDN2 in cell proliferation was caused by CLDN2/ZO1/ZONAB complex formation in the nucleus, resulting in the nuclear enhancement distribution of CLDN2.^22^ We confirmed the localisation of CLDN2 which was primarily observed at the membrane of CRC cells and not in the nucleus. The difference in localisation of CLDN2 in our experiments may arise from the different cell type used (A549 vs. Caco2 or HEK293T) and the tissue of origin. In addition, similar researches also indicated that tight junction proteins (claudins) and their adaptors (such as ZO‐1) not only stabilise the cell cytoskeleton but also form complex (such as ZO‐1/ZONAB and E‐cadherin/β‐catenin complex) to mediate signalling pathways, regulating epithelial proliferation, polarity and differentiation.^45^, ^47^ As for ZONAB, one early study had illustrated a ZONAB/DbpA complex, cooperating with symplekin, to mediate epithelial proliferation and cyclin D1 regulation.^48^ And a subsequent research group detailed CLDN2 expression as the essential mediator in the tumour‐promoting role of symplekin. The latter study suggested that symplekin cooperated with ZONAB/Dbpa to enhance CLDN2 expression, which in turn prevented the relocalisation of ZONAB away from the nucleus upon symplekin downregulation. Thus, CLDN2 and symplekin/ZONAB/DbpA complex formed a positive feedback loop to modulate the expression of downstream target gene cyclin D1.^8^ In our study, we discovered NDRG1 as the downstream target gene of CLDN2/ZO1/ZONAB axis in CRC proliferation and migration. And our study supports the needs for understanding the detailed mechanism underlying nuclear ZONAB regulation on NDRG1 transcription .

In summary, the current research illustrated the oncogenic role of CLDN2, mechanically by suppressing NDRG1 to activate a chain of downstream gene expression, finally facilitating proliferation and migration/invasion of CRC. Moreover, the study, for the first time, revealed that CLDN2 stabilised CLDN2/ZO1/ZONAB complex on the membrane and prevented ZONAB shuttle into the nucleus, suggesting CLDN2 may serve as a promising therapeutic target for CRCs.

## CONSENT FOR PUBLICATION

The content of this manuscript has not been previously published and all authors have agreed to publish this manuscript.

## COMPETING INTERESTS

The authors declare that they have no competing interests.

## Supporting information

Supporting Information
