# Supplementary material for: Claudin‐2 promotes colorectal cancer growth and metastasis by suppressing NDRG1 transcription
Source: Clin Transl Med. 2021 Dec 29;11(12):e667. doi: 10.1002/ctm2.667 (PMC8715829; doi:10.1002/ctm2.667)
Supplement: Supplementary file 1 — Supporting Information [file CTM2-11-e667-s001.docx]

**Supplementary Figures and Tables**

**
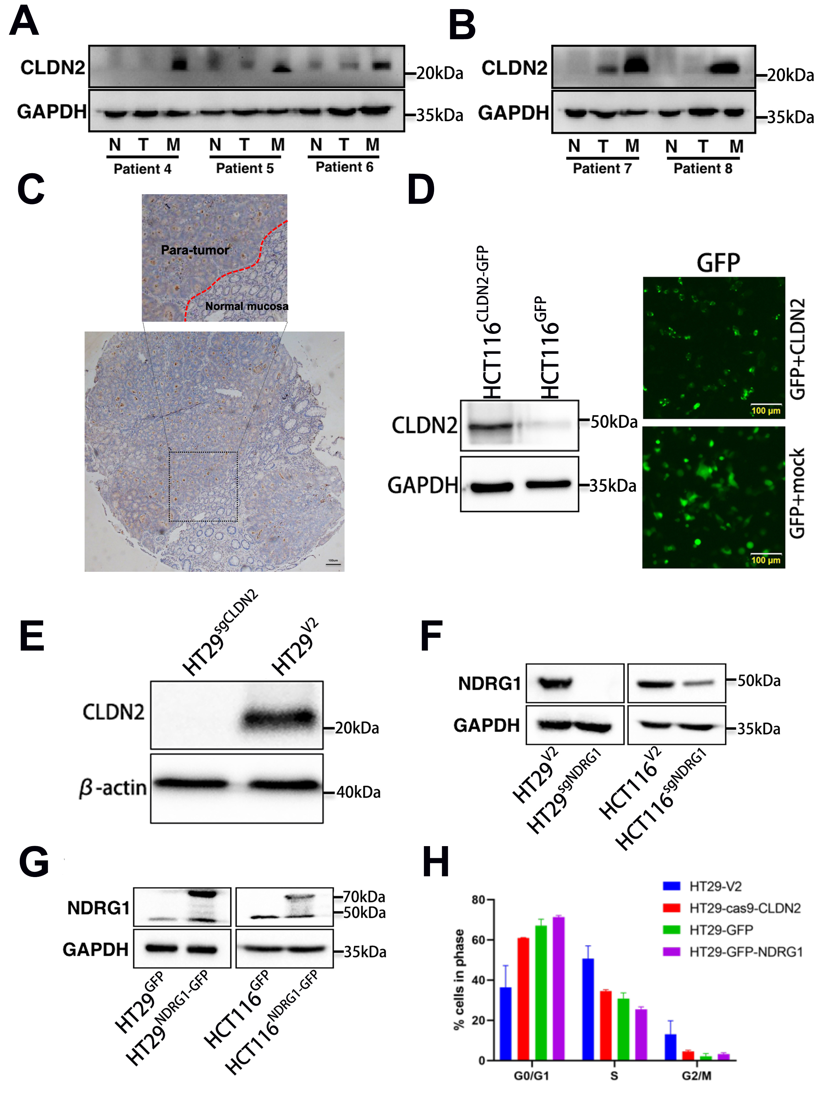
Figure S1**

**Figure S1. CLDN2 expression in CRC tissue and CRC cells**

**(A-B)** Western blot analysis of CLDN2 expression in five paired normal mucosa, colorectal cancer, and liver metastasis tissues. **(C)** Representative image of IHC staining for CLDN2 in adjacent normal mucosa and para-tumor tissue; scale bar, 100 μm. **(D)** Effect of CLDN2 overexpression by GFP-Lenti-V2 in HCT116 colon cancer cell as detected by western blot and immunofluorescence analysis; scale bar, 100 μm. **(E)** Effect of CLDN2 knockout by CRISPR-Cas9 in HT29 colon cancer cell as detected by western blot analysis. **(F)** Effect of NDRG1 knockout by CRISPR-Cas9 in HT29 and HCT116 colon cancer cells as detected by western blot analysis. **(G)** Effect of NDRG over expression by GFP-Lenti-V2 in HT29 and HCT116 colon cancer cells as detected by western blot. **(H)** Cell cycle profile was analyzed by FACS in indicated cells. The analysis was performed at least three times independently.

**
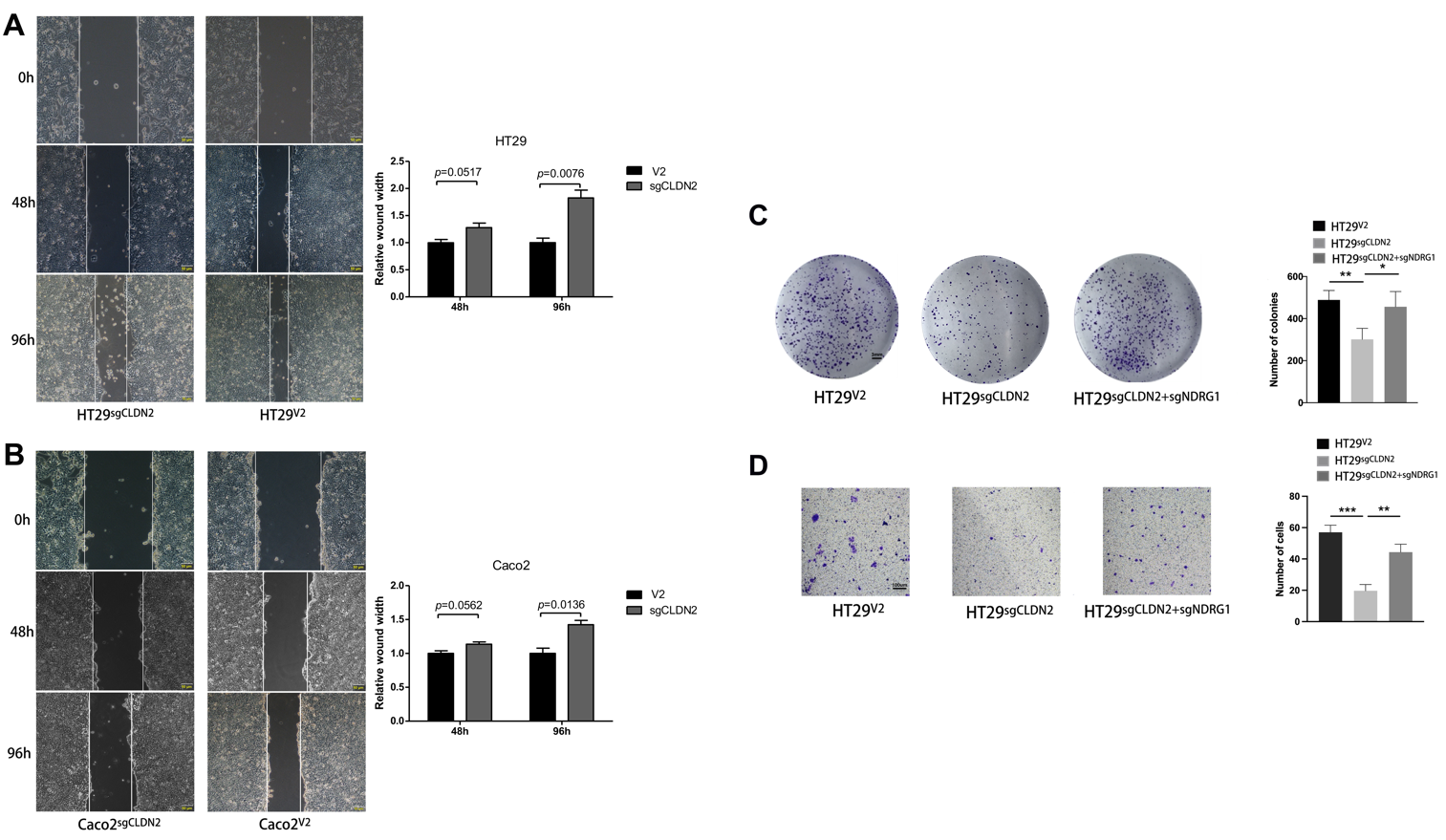
Figure S2**

**Figure S2. CLDN2 depletion attenuated CRC cell migration**

**(A-B)** Gross photos of cells wound-healing migration assay in HT29 and Caco2 cells at 0h, 48h, 96h. Left panel: CRISPR-mediated CLDN2 knockout cells; Middle panel: vector control cells; scale bar, 50 μm. And the quantification was conducted as show in right panel. **(C**) Representative colony formation images and quantification numbers of HT29 cells; scale bar, 3 mm. **(D)** Representative transwell migration images and quantification numbers of HT29 cells; scale bar, 100 μm.

**
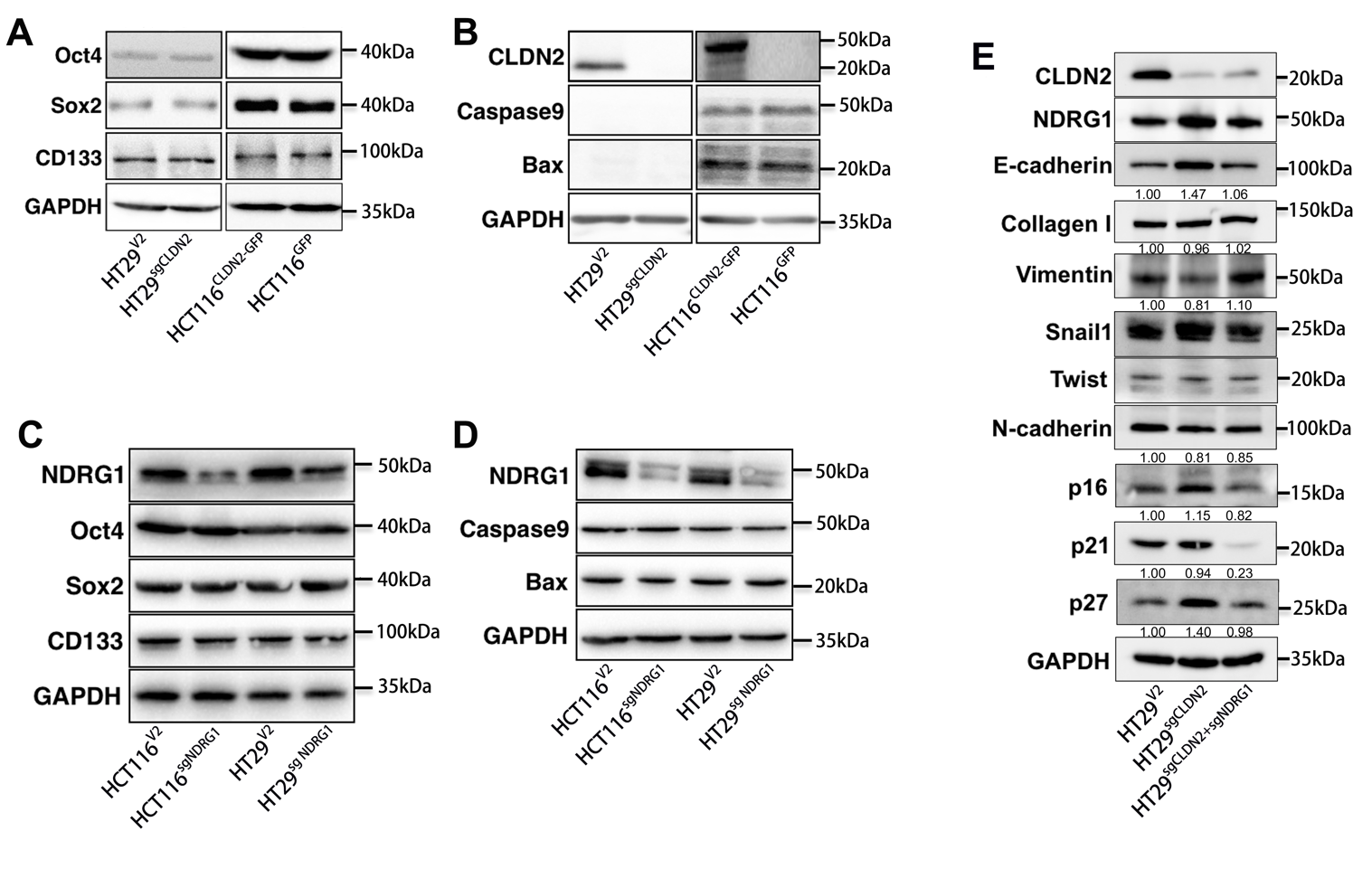
Figure S3**

**Figure S3. The expression of stemness marker and apoptosis related proteins in cells either CLDN2/NDRG1 knock-down or CLDN2 over-expression respectively**

**(A-B)** Western blot analysis of stemness and apoptosis related genes (Oct4, Sox2, CD133, Caspase9, and Bax) in CLDN2 knockout HT29 and overexpression HCT116 colon cancer cells. **(C-D)** Western blot analysis of stemness and apoptosis related genes (Oct4, Sox2, CD133, Caspase9, and Bax) in NDRG1 knockout HT29 and HCT116 colon cancer cells. **(E)** Western blot analysis of expression of indicated EMT markers (E-cadherin, Collagen I, Vimentin, Snail 1, Twist, and N-cadherin) and CDKs (p16, p21, and p27) in HT29 cells.


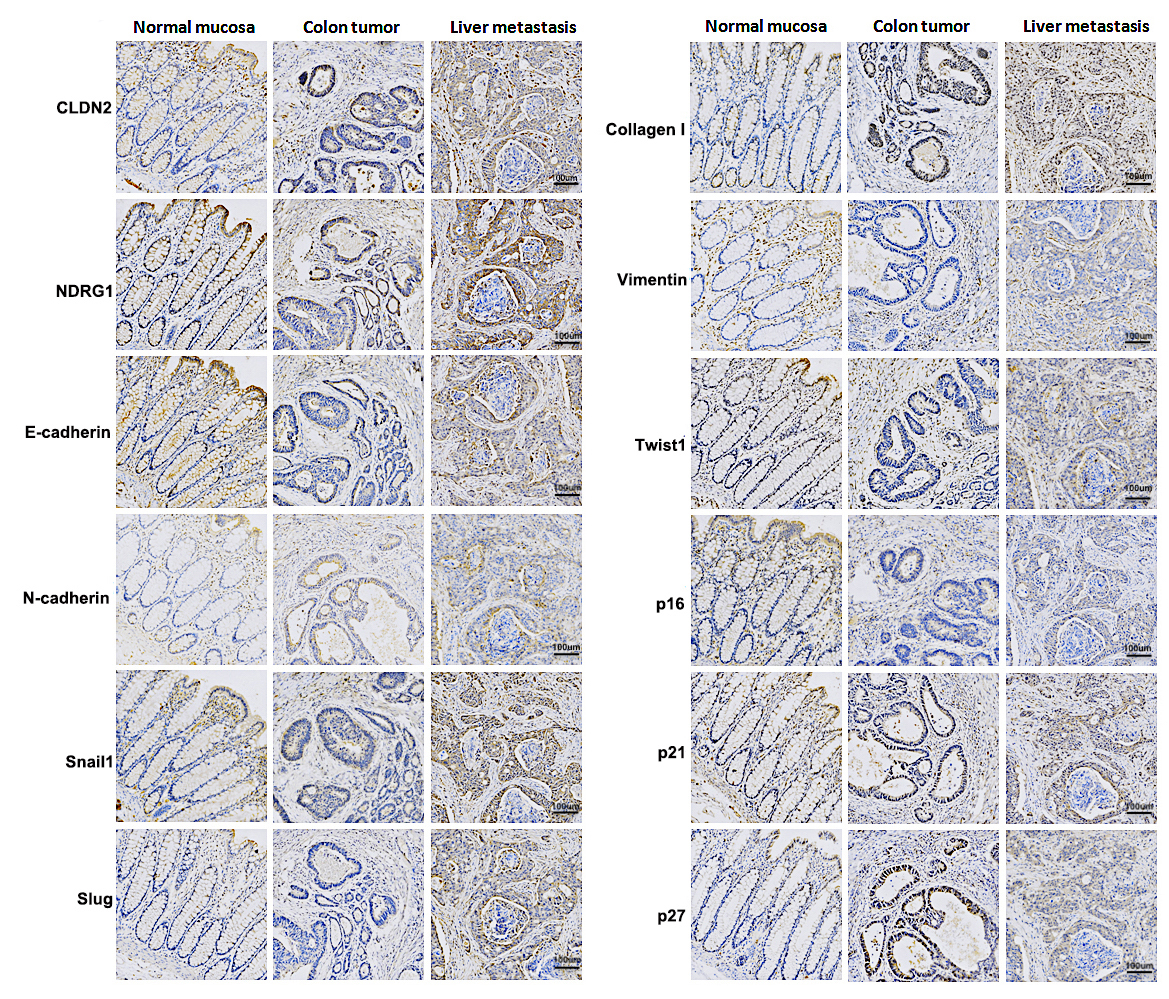
**Figure S4**

**Figure S4. The expression of EMT markers and CDK inhibitors in clinical colorectal liver metastasis tissue**

IHC analysis of representative images for CLDN2, NDRG1, EMT markers including E-cadherin, N-cadherin, Snai1, Slug, Collagen I, Vimentin, Twist1, and CDK inhibitors including p16, p21 and p27 in clinical colorectal liver metastasis tissue; scale bar, 100 μm.

**Figure S5**

**
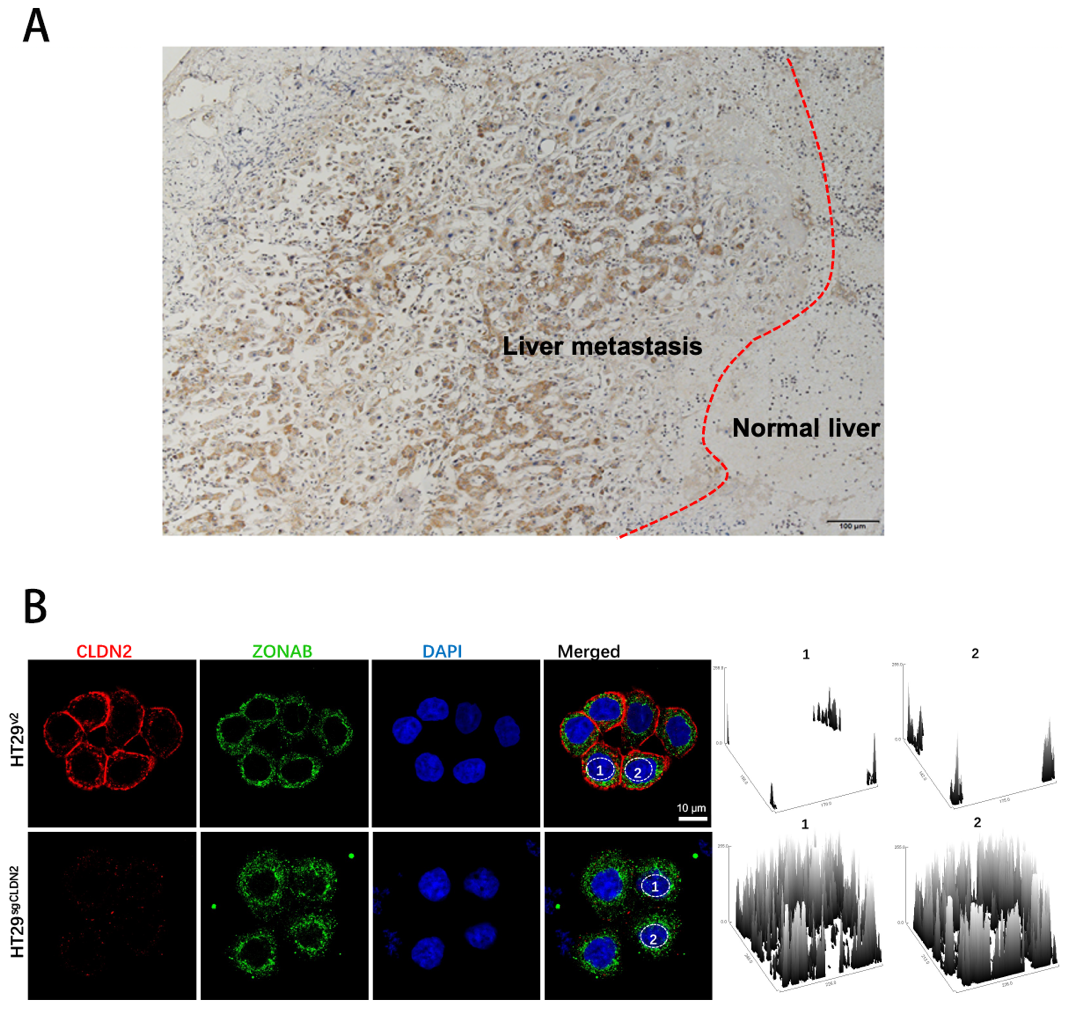
**

**Figure S5. CLDN2 expression in liver tissues and ZONAB expression in HT29 cell nuclear**

**(A)** The expression of CLDN2 in human colorectal liver metastasis tissues was detected by IHC assay using specific antibody against CLDN2. Scale bar, 100 μm. **(B)** The expression of CLDN2 and ZONAB in HT29 cells. The right two panels represented quantification of nuclear ZONAB in single cell marked as 1 and 2 respectively. Top 1 and 2 quantification represented wild type cells, while bottom 1 and 2 were CLDN2 depletion cells.

**Figure S6**

**
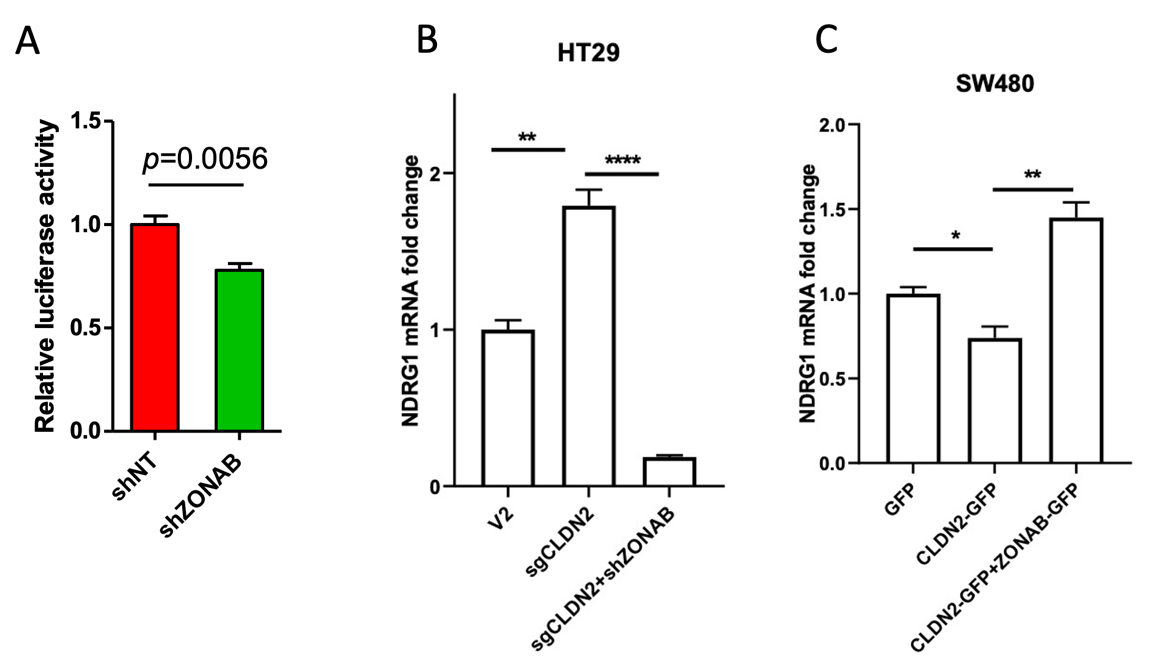
**

**Figure S6. ZONAB promotes NDRG1 transcription in CRC cells**

(A) Knocking down ZONAB resulted in NDRG1 increase examined by luciferase report assay. (B) CLDN2 depletion led to NDRG1 transcription increase while it was abolished by ZONAB knock-down in HT29 cells. (C) Overexpression of CLDN2 significantly decreased NDRG1 transcription, and ZONAB overexpression blocked CLDN2-medited NDRG1 decrease in SW480 cells. * *p* < 0.01, ** *p* < 0.01, *** *p* < 0.001 by t-test. Similar results were obtained from three independent experiments.

**Table S1 CRC cases information**

| Characteristic | Relative low level of CLDN2 (n=49) | Relative high level of CLDN2 (n=40) | P value |
| --- | --- | --- | --- |
| Age |  |  | 0.747 |
| ≥75y | 37 | 29 |  |
| ＜75y | 12 | 11 |  |
| Gender |  |  | 0.691 |
| Male | 29 | 22 |  |
| Female | 20 | 18 |  |
| T stage |  |  | 0.681 |
| T1-2 | 2 | 1 |  |
| T3-4 | 47 | 39 |  |
| N stage |  |  | 0.475 |
| N0 | 27 | 19 |  |
| N1-2 | 22 | 21 |  |
| M stage |  |  | 0.216 |
| M0 | 1 | 3 |  |
| M1 | 48 | 37 |  |
| Histologic grade |  |  | 0.763 |
| G1-2 | 35 | 36 |  |
| G3 | 4 | 4 |  |
| Presence or lymphatic vascular invasion | 0 | 2 | 0.113 |
| Presence of perineural invasion | 1 | 0 | 0.370 |
| Stage (AJCC 7th) |  |  | 0.602 |
| Stage I-II | 26 | 19 |  |
| Stage III-IV | 23 | 21 |  |

**Table S2 Antibody list**

| **Antibody** | **Manufacturer** | **Cat #** | **Dilution** |
| --- | --- | --- | --- |
| CLDN2 | Invitrogen | 32-5600 | 1:1000 |
| NDRG1 | Abclonal | A2142 | 1:1000 |
| Vimentin | Abcam | ab92547 | 1:2000 |
| E-Cadherin | Abcam | ab1416 | 1:1000 |
| Collagen1 | Abcam | ab34710 | 1:2000 |
| Fibronectin | Abcam | ab2413 | 1:3000 |
| FAP | Abcam | ab28244 | 1:2000 |
| a-SMA | Abcam | ab5694 | 1:2000 |
| Oct4 | Abcam | Ab18976 | 1:2000 |
| Sox2 | Abcam | Ab97959 | 1:2000 |
| CD133 | Bioss | bs4770R | 1:2000 |
| Capase9 | Bioss | bs0049R | 1:2000 |
| Bax | Bioss | bs0127M | 1:2000 |
| β-Tubulin | ZSGB-BIO | TA-10 | 1:5000 |
| p16 | Millipore | MAB4133 | 1:1000 |
| P21 | Millipore | 05-345 | 1:1000 |
| p27 | Millipore | 2664921 | 1:1000 |
| ZO1(WB/chip) | Invitrogen | 33-9100 | 1:1000 |
| ZONAB(WB/chip) | Invitrogen | 40-2800 | 1:1000 |
| ERK | HuaBio | RT1453 | 1:1000 |
| p-ERK | HuaBio | RT1206 | 1:1000 |
| STAT3 | HuaBio | RT1591 | 1:1000 |
| p-STAT3 | HuaBio | ET1603-40 | 1:1000 |
| actin | ZSGB-BIO | TA-09 | 1:5000 |
| Peroxidase-Conjugated Goat anti-Mouse IgG(H+L) | ZSGB-BIO | ZB-2305 | 1:5000 |
| Peroxidase-Conjugated Goat anti-Rabbit IgG(H+L) | ZSGB-BIO | ZB-2301 | 1:5000 |
| H3 | CST | 2650S | 1:2000 |
| H3 | Millipore | 06-755 | 1:2000 |
| IgG | CST | 2729 | 1:1000 |
| GAPDH | ZSGB-BIO | TA-08 | 1:5000 |

**Table S3** **List of Oligo and Primers**

CRISPR-Cas9 Oligo sequence

CLDN2-F 5-CACCGCTAGGATGTAGCCCACAAGT-3

CLDN2-R 5-AAACACTTGTGGGCTACATCCTAGC-3

NDRG1-F 5-CACCGGGCGCAGCCTCCTTCCCCGC-3

NDRG1-R 5-AAACGCGGGGAAGGAGGCTGCGCCC-3

Primer sequence

pLenti-EGFP-FP:

GGAGGTTCCGGTGGAAGCGGAGGTAGCGGCGGATCCATGGTGAGCAAGGGCGAG

pLenti-EGFP-RP:

GGTGGAGCCTGCTTTTTTGTACAAACTTG

pLenti-cmv-GFP-Claudin2-FP:

ACAAAAAAGCAGGCTCCACC ATGGCCTCTCTTGGCCTCC

pLenti-cmv-GFP-Claudin2-RP:

CCGCTTCCACCGGAACCTCCCACATACCCTGTCAGGC

pCMV-C-EGFP-NDRG1-FP:

TCCAAGCTTATGTCTCGGGAGATGCAGGATG

pCMV-C-EGFP-NDRG1-RP:

TCTGTCGACGCAGGAGACCTCCATGGACTTG

NDRG1-promoter primer-FP:

GTTGCGCAAGCAGTCGTAG

NDRG1-promoter primer-RP:

GCCCATACGGAGACACAAGG

pCMV-C-EGFP-ZONAB-FP:

TGGTCCAAACCAGCCGTCTGTT

pCMV-C-EGFP-NDRG1-RP:

GTTCTCAGTTGGTGCTTCACCTG

**Supplementary materials and methods**

**Western blot analysis**

Western blot analysis was performed as described previously [1]. Briefly, protein lysates (usually 20 μg) were fractionated by 8% or 10% sodium dodecyl sulphate polyacrylamide gel (SDS-PAGE), and incubated with a primary antibody with a diluted concentration based on its protocol (Table S1). β-Actin or GAPDH were used as sample loading controls.

**Cell cycle Profiling**

Cell cycle distribution was detected by flow cytometry. HT 29 cells (1-6 × 10^6^ cells/well) were seeded in 6 well plate. Cells were collected 48-72 hours post-transfection and washed with PBS, and then fixed in 70% ethanol overnight at 4 °C. And then, cells were incubated with RNase A for 30 min at 37 °C and stained with PI for 30 min at 4 °C in dark. We examined the cells using a flow cytometer (BD Pharmingen, USA) and cell population was analyzed by using the Mod Fit LT software (Verity Software House, Topsham, ME).

**Wound-healing migration assay**

Cells were cultured until confluent on the 6-well plates and the cell monolayer was scraped by a pipette tip in a straight line. The detached cells and debris were removed by washing with PBS buffer. Subsequently, images of the scratched area were taken at 0, 48 and 96 h by phase-contrast microscopy (Olympus, Shinjuku, Tokyo, Japan).
